# Improved Natural Killer cell activity and retained anti-tumor CD8^+^ T cell responses contribute to the induction of a pathological complete response in HER2-positive breast cancer patients undergoing neoadjuvant chemotherapy

**DOI:** 10.1186/s12967-015-0567-0

**Published:** 2015-06-27

**Authors:** E Muraro, E Comaro, R Talamini, E Turchet, G Miolo, S Scalone, L Militello, D Lombardi, S Spazzapan, T Perin, S Massarut, D Crivellari, Riccardo Dolcetti, D Martorelli

**Affiliations:** Cancer Bio-Immunotherapy Unit, Department of Translational Research, CRO Aviano, IRCCS, National Cancer Institute, Via F. Gallini 2, 33081 Aviano, PN Italy; Unit of Epidemiology and Biostatistics, CRO Aviano, IRCCS, National Cancer Institute, Via F. Gallini 2, 33081 Aviano, PN Italy; Scientific Direction, CRO Aviano, IRCCS, National Cancer Institute, Via F. Gallini 2, 33081 Aviano, PN Italy; Department of Medical Oncology, CRO Aviano, IRCCS, National Cancer Institute, Via F. Gallini 2, 33081 Aviano, PN Italy; Department of Pathology, CRO Aviano, IRCCS, National Cancer Institute, Via F. Gallini 2, 33081 Aviano, PN Italy; Division of Breast Surgical Oncology, CRO Aviano, IRCCS, National Cancer Institute, Via F. Gallini 2, 33081 Aviano, PN Italy

**Keywords:** Breast cancer, Neoadjuvant chemotherapy, Antitumor immunity, CD8^+^ T lymphocytes, NK cells, Immunomonitoring, Polyfunctional T cell responses, Th17 cells, HER2-overexpression, Pathological complete response

## Abstract

**Background:**

Locally advanced HER2-overexpressing breast cancer (BC) patients achieve a high rate of pathological complete responses (pCR) after neoadjuvant chemotherapy (NC). The apparently unaltered immune proficiency of these patients together with the immune-modulating activities of NC drugs suggest a potential contribution of host immunity in mediating clinical responses. We thus performed an extensive immunomonitoring in locally advanced BC patients undergoing NC to identify immunological correlates of pCR induction.

**Methods:**

The immune profile of 40 HER2-positive and 38 HER2-negative BC patients was characterized at diagnosis and throughout NC (Paclitaxel and Trastuzumab, or Docetaxel and Epirubicin, respectively). The percentages of circulating immune cell subsets including T and B lymphocytes, Natural Killer (NK) cells, regulatory T cells, T helper 17 lymphocytes, were quantified by multiparametric flow cytometry. NK cells functional activity was evaluated through the analysis of NF-kB nuclear translocation by Multispectral flow cytometry, and with the in vitro monitoring of Trastuzumab-mediated antibody-dependent cell cytotoxicity (ADCC). CD8^+^ T cell responses against six different tumor-associated antigens (TAA) were characterized by IFN-γ ELISPOT and IFN-γ/IL-2 DualSpot assays.

**Results:**

After NC, HER2-positive patients showed a significant increase in the number of NK cells and regulatory T cells irrespective of the pathological response, whereas patients undergoing a pCR disclosed higher percentages of T helper 17 cells. Notably, a significant increase in the number of activated NK cells was observed only in HER2-positive patients achieving a pCR. Characterization of anti-tumor T cell responses highlighted sustained levels of CD8^+^ T cells specific for survivin and mammaglobin-A throughout NC in patients undergoing a pCR in both arms. Moreover, HER2-positive patients achieving a pCR were characterized by a multi-epitopic and polyfunctional anti-tumor T cell response, markedly reduced in case of partial response.

**Conclusions:**

These results indicate that maintenance of functional T cell responses against selected antigens and improvement of NK cell proficiency during NC are probably critical requirements for pCR induction, especially in HER2-positive BC patients.

Trail registration: Trial registration number: NCT02307227, registered on ClinicalTrials.gov (http://www.clinicaltrials.gov, November 26, 2014).

**Electronic supplementary material:**

The online version of this article (doi:10.1186/s12967-015-0567-0) contains supplementary material, which is available to authorized users.

## Background

Breast cancer (BC) is characterized by a complex biological heterogeneity, also reflected in the clinical setting in which distinct tumor subtypes show different rates of pathological complete response (pCR) induction after neoadjuvant chemotherapy (NC). The highest pCR odds are achieved in patients with triple negative (TN) or HER2-positive/hormone receptor-negative BCs [1]. Neoadjuvant therapy trials provide an ideal platform to identify biomarkers of possible predictive and/or prognostic significance, and pCR thus represents an endpoint for the rapid triage of drugs that may be helpful for subsequent adjuvant purposes [2].

In locally advanced BC patients treated with NC, the content of Tumor Infiltrating Lymphocytes (TILs) in the primary biopsy was shown to predict pCR [3, 4], especially in the TN and HER2-positive subsets [5, 6]. In these patients, taxane-based NC was shown to increase the number of tumor infiltrating CD8^+^ T cells [7, 8] and to induce their activation through the expression of Granzyme B [9]. Notably, a pronounced lymphocytic infiltration observed after treatment correlated with an improved outcome [8].

Besides playing an important role in tumor surveillance and modulation of tumor growth [10, 11], innate and adaptive immunity may also be involved in the response to chemotherapy as suggested by several trascriptomes analyses of mammary carcinomas [12]. Indeed, the destruction of tumor cells by chemotherapeutic agents may release tumor-associated antigens (TAAs), which, in turn, can trigger immune responses against tumor cells. This immunotherapeutic effect induced by chemotherapy may be particularly strong in patients already spontaneously sensitized against tumor antigens, thus potentially leading to a pCR [13, 14]. Notably, innate and adaptive immune mechanisms are emerging as key players also in the modulation of the activity of HER2-targeted drugs, such as the monoclonal antibody (moAb) Trastuzumab [5]. Indeed, higher efficiency of Antibody Dependent Cell Cytotoxicity (ADCC) and Natural Killer (NK) cell lysis were reported in clinical responders to Trastuzumab if compared with non-responders [15, 16]. Interestingly, the efficacy of Trastuzumab treatment was associated with the enhanced in situ infiltration of interferon-γ producing CD8^+^ T cells [17–19] and CD4^+^ T helper (Th) lymphocytes [20], and decreased numbers of circulating regulatory T cells (Treg)/CD4^+^ [21] and reduced Treg/inflammatory Th17 ratios [22].

In agreement with these findings, our recent characterization of the immune profile of 61 locally advanced BC patients eligible for a NC schedule demonstrated that, at diagnosis, patients with HER2-overexpressing cancers had a retained immune proficiency and higher CD8^+^ T cell responses against several TAAs if compared to HER2-negative cases, whose general immune background, on the contrary, appeared compromised [23].

In the present study, we report on the results of the phenotypic and functional characterization of circulating immune cells in the same cohort of BC patients throughout NC treatment, based on the use of Paclitaxel and Trastuzumab in the HER2-positive arm and Epirubicin and Docetaxel in HER2-negative patients. Our main objective was to gain insights on the possible contribution of the host immune system to the clinical outcome of these patients, taking advantage of the absence of anthracyclines-related toxicity [24]. We selected peripheral blood as the easiest source of biological samples and thus as the ideal candidate for our immunomonitoring analysis.

Herein we provide evidence indicating that, compared to partial responders, pCR patients were characterized by a more efficient functionality of both innate and adaptive immune cells, especially among HER2-positive patients. In particular, our results are consistent with a relevant contribution of circulating NK cells and TAA-specific CD8^+^ T cells in inducing a pCR, at least in the HER2-positive arm.

## Methods

### Patients assessments and therapy

This study, part of a phase II mono-institutional trial, included the analyses performed in blood samples obtained from 78 patients with histologically confirmed locally advanced breast carcinoma (defined as not susceptible of conservative surgery at diagnosis; UICC, International Union Against Cancer, stage II to III). HER2 status was assessed by immunohistochemistry (IHC) and chromogenic in situ hybridization (CISH) or fluorescence in situ hybridization (FISH) in case of IHC 2+. All patients had the following clinical features: Eastern Cooperative Oncology Group performance status of 0 or 1; baseline left ventricular ejection fraction measured by ultrasonography greater than 50%; adequate organ function (bone marrow function: neutrophils ≥2.0 × 10^9^/L, platelets ≥120 × 10^9^/L; liver function: serum bilirubin <1.5 times the upper limit of normal (ULN), transaminases <2.5 times ULN, alkaline phosphatase ≤2.5 times ULN, serum creatinine <1.5 times ULN).

Patients with HER2-overexpressing locally advanced BC (n = 40; median age of 46 years, range 23–70) received an anthracycline-free NC with Trastuzumab (loading dose 4 mg/kg intravenously, then 2 mg/kg weekly) and concomitant weekly Paclitaxel (80 mg/m^2^; [TP]) for 3 cycles, followed by evaluation and, in case of clinical response, 3 more cycles to obtain a pCR. The HER2-negative breast cancer group of patients (n = 38; median age of 46 years, range 27–69) was treated with a NC regimen containing both taxanes and anthracyclines (8 cycles of Docetaxel [75 mg/m^2^] and concomitant Epirubicin [90 mg/m^2^] every 3 weeks [ED]). According to RECIST criteria a total of 17 pCR (42.5%) were achieved in the HER2-positive arm and 5 (13.2%) within the HER2-negative group. After NC completion, patients underwent primary surgery (mastectomy or conservative treatment) as well as axillary node dissection. The breast conserving surgery rate was 37.5% (15/40) in HER2-positive patients and 42.1% (16/38) in HER2-negative.

Patients with progressive disease after the first evaluation (12 weeks of treatment) underwent primary surgery and received adjuvant chemotherapy using different regimens according to international guidelines. Instrumental evaluation was performed at baseline and every 12 weeks. This study (ClinicalTrials.gov Identifier: NCT02307227) was conducted according to the ethical principles of the Declaration of Helsinki and approved by the local Ethical Committee (Comitato Etico Indipendente del CRO di Aviano, May 29, 2006). Written informed consent was obtained from all patients and donors.

### Sample collection

Blood and serum samples were collected from each patient at diagnosis and throughout NC, after 12 and 24 weeks of treatment, and transported at room temperature. Peripheral blood mononuclear cells (PBMCs) were freshly isolated (within 5 h after blood drawing) from heparinised blood of patients by Ficoll-Hypaque gradient (Lymphoprep, Fresenius Kabi Norge Halden) using standard gradient separation. Cells were washed in PBS (Biomerieux), counted using Trypan blue (viability >90%) and viably frozen (90% heat-inactivated Fetal Bovine Serum [FBS; Gibco^®^, Life Technologies] and 10% DMSO) at −80°C for 24 h and then in liquid nitrogen until use. After thawing in RPMI-1640 medium (Sigma-Aldrich) with 3 μg/ml Deoxyribonuclease (Sigma-Aldrich), cells were washed in PBS (Biomerieux) and counted again to check viability (>80%). We included also 20 age-matched healthy women as controls, whose PBMCs were collected from buffy coat products and stored as described above. Patient’s and donor’s samples were genotyped to identify those expressing the allele HLA-A*0201 by performing PCR sequencing based typing with specific primers [25].

### Peptide selection and synthesis

The following 13 immunogenic HLA-A*0201-restricted nonamer (9-mer) peptides, derived from different TAA were selected for the study: Mammaglobin-A LIY_83-92_ LIYDSSLCDL, Survivin ELT_95-104_ ELTLGEFLKL, Survivin LDR_104-113_ LDRERAKNKI, Mucin-1 LLL_12-20_ LLLLTVLTV, Mucin-1 STA_950-958_ STAPPVHNV, Her2/neu KIF_369-377_ KIFGSLAFL, Her2/neu CLT_789-797_ CLTSTVQLV, Her2/neu VLV_851-859_ VLVKSPNHV, Her2/neu ELV_971-979_ ELVSEFSRM, Trag-3 HAC_37-45_ HACWPAFTV, Trag-3 SIL_57-66_ SILLRDAGLV, Bcl-xL RIA_165-174_ RIAAWMATYL, and Bcl-xL YLN_173-182_ YLNDHLEPWI. The HLA-A*0201-restricted Flu matrix 1 (M1) GIL_58-66_ peptide (GILGFVFTL) was used as the positive control in the IFN-γ ELISPOT assays, while the following mix of peptides derived from Cytomegalovirus- (CMV), Epstein-Barr Virus- (EBV), and Flu antigens was the positive control in dual color IFN-γ/IL-2 ELISPOT assays: CMV pp65 IPS_113-121_ IPSINVHHY, CMV pp65 TPR_417-426_ TPRVTGGGAM, EBV BMLF1 GLC_259-267_ GLCTLVAML, EBV EBNA3B IVT_416-424_ IVTDFSVIK, EBV BRLF1 ATI_134-142_ ATIGTAMYK, Flu M1 GIL_158-66_ GILGFVFTL, Flu NucleoProtein (NP) RVL_342-351_ RVLSFIKGTK, Flu M1 SII_13-21_ SIIPSGPLK, Flu NP ELR_380-388_ ELRSRYWAI. All peptides were produced by fluorenylmethoxycarbonil synthesis (Primm) and purity (>95%) was determined by reverse-phase high-performance liquid chromatography and verified by mass spectral MALDI-TOF analysis. Peptides were dissolved in DMSO at a concentration of 2.5 mg/ml and stored at −80°C until use. Work stocks for each peptide were prepared in PBS at a final concentration of 500 μg/ml and stored frozen.

### Flow cytometry and multispectral imaging

The following fluorescent-conjugated monoclonal antibodies were used: α-CD3 fluorescein isothiocyanate (FITC) or phycoerythrin-texasred (ECD; mouse immunoglobulin (Ig) G1, clone UCHT1), α-CD4 phycoerythrin-cyanine5 (Pe-Cy5; mouse IgG1, 13B8.2), α-CD8 phycoerythrin-cyanine7 (Pe-Cy7; mouse IgG1, SFCI2IThy2D3), α-CD16 FITC (mouse IgG1, 3G8), α-CD19 FITC (mouse IgG1k, J3-119), α-CD25 ECD (mouse IgG2a, B1.49.9), and α-CD45RA ECD (mouse IgG1, 2H4LDH11LDB9) all from Beckman Coulter; α-CD56 phycoerythrin (PE; mouse IgG1k, B159) and α-CD197 PE (CCR7, rat IgG2a k, 3D12) purchased from BD Biosciences (Becton–Dickinson); α-CD4 Pe-Cy7 (mouse IgG1 k, RPA-T4), α-CD127 Pe-Cy5 (mouse IgG1, eBioRDR5), α-FoxP3 PE (Rat IgG2a k, PCH101), and α-Interleukin (IL) 17 FITC (mouse IgG1k, eBio64DEC17) from eBioscience; α-NF-κB p65 FITC (mouse IgG1, F-6) purchased from Santa Cruz Biotechnology, INC. DRAQ5™ fluorescent DNA dye from BioStatus Limited was used in nuclear localization analysis. Properly labelled isotypic antibodies were used as negative controls. All antibodies were used in an appropriate volume of 10% rabbit serum (Dako) and PBS to reduce nonspecific signal. Intracellular FoxP3 was determined using the eBioscience FoxP3 Staining Buffer Set (eBioscience) according to the manufacturer’s instructions. Briefly, after surface molecules staining, cells were fixed and permeabilized with fixation/permeabilization buffer for 30 min at 4°C, washed twice, and labelled with FoxP3 antibody in the presence of 2% rabbit serum in PBS at 4°C for at least 30 min and, after two washes, cells were re-suspended in PBS with 1% paraformaldehyde. To evaluate IL-17 release, cells were pretreated with 25 ng/ml Phorbol 12-Myristate 13-Acetate (PMA, Sigma-Aldrich) and 1 μg/ml Ionomycin (Sigma-Aldrich) in the presence of 500 ng/ml Monensin-A (Sigma-Aldrich) in complete medium (RPMI-1640 [Sigma-Aldrich] supplemented with 10% FBS) for 4 h at 37°C. Cells were labelled for surface molecules, then fixed with 2% paraformaldehyde in complete medium for 10 min at room temperature, washed in complete medium and permeabilized using 90% methanol diluted in H_2_O. After 10 min incubation in ice, cells were washed in permeabilization buffer (PBS with 0.5% Bovine Serum Albumin [BSA; Sigma-Aldrich]) and stained with 0.125 μg of α-IL-17 antibody in 2% rabbit serum diluted in permeabilization buffer at 4°C for 30 min. Samples were washed twice and re-suspended in PBS with 1% paraformaldehyde for flow cytometry analysis. At least 5 × 10^4^ cells for surface markers and 1 × 10^6^ cells for intracellular staining were acquired. Cytofluorimetric analysis was performed with a Cytomics FC500 (Beckman Coulter, Fullerton, CA, USA), photomultiplier voltages and compensation were set with unstained and stained cells. Data were analyzed with CXP (Beckman Coulter, Fullerton, CA, USA) and FlowJo (Tree Star, Ashland, OR, USA) softwares.

Immune cells subsets were identified as following (see Additional file 1): CD3^+^ T cells, CD19^+^ B cells, CD3^−^CD16^+^CD56^+^ NK cells, CD3^+^CD4^+^ T cells, CD3^+^CD8^+^ T cells, CD3^+^CD4^+^CD25^+^CD127^−/low^FoxP3^+^ Treg cells, CD3^+^CD4^+^IL17^+^ Th17 cells. Among CD3^+^CD4^+^ and CD3^+^CD8^+^ T cells, CCR7^+^CD45RA^+^ naïve T cells (T_naive_), CCR7^+^CD45RA^−^ Central Memory T (T_CM_) cells, CCR7^−^CD45RA^−^ Effector Memory T cells (T_EM_), CCR7^−^CD45RA^+^ Terminally Differentiated T cells (T_Temra_) were identified.

Purified NK cells were obtained by immunomagnetic enrichment protocols using the human NK Cell Isolation Kit (Miltenyi Biotec) according to manufacturer’s instructions.

To determine NF-κB nuclear internalization, purified NK cells (1.5 × 10^6^) were labeled with α-NF-κB FITC and α-CD56 PE monoclonal antibodies. Briefly, after CD56 staining in 10% rabbit serum, cells were fixed and permeabilized as described above at 4°C, washed twice, and labeled with NF-κB antibody (1:100) in PBS 2% rabbit serum for 45 min at 4°C. After two washes in PBS/0.5% BSA, cells were re-suspended in PBS with 1% paraformaldehyde and DRAQ5 DNA dye (5 µM). Cells were run on the ImageStreamX cytometer using the INSPIRE software (Amnis Corporation, Seattle, WA, USA) and images were analyzed using the IDEAS software (Amnis Corporation, Seattle, WA, USA). Cells were excited using a 488 nm laser with intensity of 40 mW. Brightfield, side scatter, fluorescent cell images were acquired at 40× magnification. Only events with brightfield areas greater than 20 μm^2^ (excluding debris) and non-saturating pixels were collected. NF-κB nuclear localization was measured using an IDEAS software feature, the Similarity Score (SS), that defines/quantifies the similarity of the nuclear (DRAQ5) and labelled-transcription factor staining patterns (NF-κB FITC). All events showing a positive SS were considered with high similarity between NF-κB and DRAQ5, thus indicating a nuclear localization of the transcription factor. Only viable cells were selected on the basis of morphologic features. Single-stained compensation controls were used to compensate fluorescence between channel images on a pixel-by-pixel basis.

### ADCC assay

The monoclonal antibody Trastuzumab acts through widely described immune-mediated mechanisms as the ADCC, which involve host’s immune cells [26]. The ADCC efficiency was evaluated in a Calcein-AM release assay, using the HER2/*neu*-overexpressing BC cell line MDA-MB453 as target cells, and patients’ PBMCs as effectors. The cell line was cultured in DMEM (Sigma-Aldrich), containing 2 mM l-glutamine, 10% FBS, 100 μg/ml streptomycin and 100 IU/ml penicillin (Sigma-Aldrich), at 37°C in 5% of CO_2_. One million target cells in exponential growth was labelled with 7.5 μM Calcein-AM (Molecular Probes) for 30 min at 37°C, washed 3 times, then incubated with Trastuzumab antibody (20 μg/ml; Roche) 1 h in ice. Without washing the persistence of soluble antibody, 1 × 10^4^ labelled-target cells per well were seeded into 96-well U-bottom plates. Experiments were conducted in triplicates at two effector (PBMCs):target ratios of 30:1 and 15:1, in 200 μl of Hank’s Balanced Salt Solution (HBSS) containing 5% FCS. After 4 h at 37°C and 5% CO_2_ the release of Calcein (excitation = 485 nm; emission = 530 nm) was measured with a fluorescence plate reader (SpectraFluorPlus, Tecan, Männedorf, Switzerland). Maximal and spontaneous Calcein release values were obtained by adding either 100 μl Lysis buffer (NaBO_3_ 0.025 M, Triton X-100 0.1%, pH 9) or HBSS, to wells containing 1 × 10^4^ labeled target cells. The percentage of lysis was calculated according to the standard formula = 100 × (experimental release − spontaneous release)/(maximal release − spontaneous release). The percentage of lysis was normalized for 10,000 NK cells using the following formula = (percentage of lysis × 10^4^)/(effector: target ratio × target cell no. in an experimental well × NK percentage in PBMCs) [27].

### IFN-γ and IFN-γ/IL-2 ELISPOT assay

The interferon (IFN)-γ release enzyme-linked immunosorbent spot (ELISPOT) assay was performed using a commercial kit (Human IFN-γ ELISPOT; Thermo scientific), according to manufacturer’s instructions. Briefly, the assay was carried out using autologous peptide-pulsed monocytes as antigen presenting cells (APCs) and isolated CD8^+^ T lymphocytes as responders. Monocytes, isolated by a 2 h plastic adherence step from patient’s PBMCs, were loaded with 10 μg/ml of each 9-mer peptide in complete medium, supplemented with 5 μg/ml of human β2-microglobulin (Sigma-Aldrich), and incubated for 2 h at 37°C with 5% CO_2_. Purified effectors were obtained by immunomagnetic enrichment protocols using the human CD8^+^ T cell isolation kit II (Miltenyi Biotec), and then cultured with peptide-loaded monocytes (50,000 cells/well) at 1:1 effector:target ratio. FLU M1_58-66_ and unstimulated monocytes were used as positive and negative controls, respectively. Cells were seeded onto ELISPOT capture plates in triplicates and incubated for 48 h at 37°C with 5% CO_2_.

IFN-γ and IL-2 dual color ELISPOT assay (TriSpot Human IFN-γ/IL-2 ELISPOT kit, Endogen) was carried out by using PBMCs derived from 4 HLA-A*0201 BC patients. Briefly, PBMCs (200,000 cells/well) were plated and stimulated for 28 h, at 37°C and 5% CO_2_, with 4 µg/ml of a panel of 4 out of the 13 TAA-derived peptides, previously validated by single color ELISPOT. The CEF mix was also used as positive control peptide stimuli. Media and PBMCs alone were used as negative controls, and the anti-CD3 antibody (clone UCHT1; BD Pharmingen™) was used as a positive control stimulus.

All plates were evaluated by a computer-assisted ELISPOT reader (Eli.Expert, A.EL.VIS GmbH, Hannover, Germany; see Additional file 2 for representative wells pictures). The number of spots in negative control wells (range 0–5 spots) was subtracted from the number of spots in stimulated wells. Responses were considered significant if a minimum of five IFN-γ or IL-2 producing cells were detected in the wells.

### Statistical methods

Data obtained from multiple independent experiments were expressed as mean and standard deviation for immunophenotypic analysis, ADCC assay, and NF-κB nuclear translocation experiments. Mean values were reported in IFN-γ and IFN-γ/IL-2 dual spot ELISPOT assays. Raw data can be provided per request. The Student’s t test for two tailed distributions and paired data was used for the statistical analysis of variations during NC in circulating immune cells percentages, NF-κB nuclear translocation levels, and percentages of lysis in ADCC assays, comparing data obtained at diagnosis with data measured respectively after 12 and 24 weeks of treatment. The Student’s t test for two tailed distributions and unpaired data was used to compare data regarding HER2-positive and HER2-negative arms, or patients achieving a pCR with partial responders within the single arms. Differences were considered statistically significant when P ≤ 0.05.

### Laboratory environment

These studies were conducted in a laboratory that operates under exploratory research principles, using established laboratory protocols, and validated assays. Moreover, the laboratory gained the ISO 9001:2008 certification (Bureau Veritas agency) with the fulfillment of the ISO 9001:2008 accreditation criteria both for research and diagnostic procedures. The date of the last audit was January 13th, 2015.

## Results

### NC induced a reshuffle of circulating immune cells in HER2-positive and HER2-negative breast cancer patients

**Figure 1:**
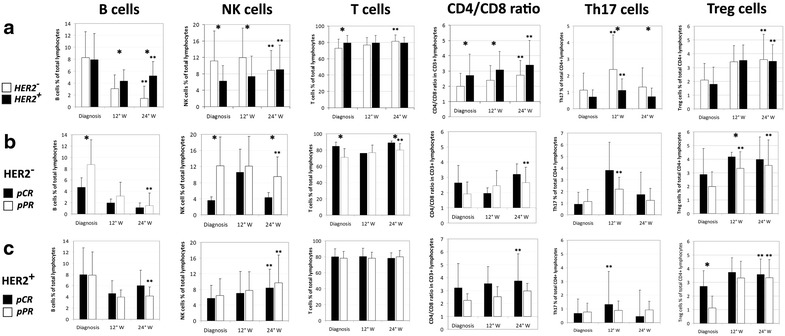
Phenotyping of circulating immune cells in HER2-negative and HER2-positive BC patients during NC. **a** Comparison of the percentage of several immune cell subsets quantified in the peripheral blood of HER-negative (*white plots*; n = 33) and HER2-positive (*black plots*; n = 23) BC patients throughout NC. *p < 0.05 comparing HER2-positive with HER2-negative patients. **b** Analysis of immune cell percentages within HER2-negative patients comparing individuals achieving a pathological complete response (pCR; *black plots*; n = 4) with those characterized by a pathological partial response (pPR; *white plots*; n = 29). *p < 0.05 comparing pCR with pPR patients. **c** Immune cell percentages observed in HER2-positive patients undergoing a pCR (*black plots*; n = 11) and in HER2-positive patients showing a pPR (*white plots*; n = 12). *p < 0.05 comparing pCR with pPR patients. *NK* Natural Killer cells, *Th17* T helper 17 cells, *Treg* regulatory T cells, *W* week, *pCR* pathological complete response, *pPR* pathological partial response; **p < 0.05 in respect to the corresponding diagnosis values.

Our previous findings showed that, at diagnosis, locally advanced BC patients with a HER2-overexpressing malignancy disclosed several differences in the distribution of circulating immune cells in comparison to HER2-negative BCs [23]. We thus wondered whether this different immune background might have influenced the response to NC and how chemotherapy treatment could affect immune cells distribution. In both groups of patients, the NC treatment induced a decrease in the number of circulating B cells, more pronounced in HER2-negative cases, an increase in the CD4/CD8 ratio, and a progressive enlargement of the Treg cell compartment preceded by a parallel increase in the number of Th17 cells after 12 weeks of treatment. As compared to the HER2-negative group, HER2-positive cases showed lower numbers of Th17 cells during NC, lower percentages of NK cells and a higher CD4/CD8 ratio at week 12, and higher numbers of CD4^+^ T_naive_ cells at the end of NC (Figure 1a and see Additional file 3). HER2-negative patients disclosed an increase in the percentage of T cells that, at the end of therapy, reached the levels observed in HER2-positive cases. Characterization of T cell memory phenotype revealed a raise in CD4^+^ T_naive_ cells in both groups, balanced by a decrease of CD4^+^ T_Temra_ cells in the HER2-positive group, and by a reduction of CD4^+^ T_EM_ cell compartment in HER2-negative cases. Among CD8^+^ T cells, T_CM_ decreased in both arms, while an increase in T_naive_ appeared only in HER2-positive patients. NK cells displayed an opposite behavior in the 2 groups, with a significant increase in HER2-positive patients during TP therapy and an important drop in ED-treated HER2-negative patients (Figure 1a and see Additional file 3).

Possible correlations between the pathological response and changes in the proportions of circulating immune cells were then investigated in both groups of BC patients. Within the HER2-negative arm, correlations with the pathological response were already highlighted at diagnosis, with pCR patients showing lower numbers of B cells and NK cells, and higher percentages of T cells compared to partial responders (pPR). Differences in the number of T lymphocytes and NK cells were maintained till the end of ED therapy. A higher percentage of Treg was also documented in pCR cases compared to partial responders after 12 weeks of NC. Interestingly, no significant changes were found in pCR patients throughout NC, while partial responders at the end of ED therapy showed decreased numbers of B cells and NK cells and an increase in T cells and CD4/CD8 ratio. In these patients, the number of Treg cells underwent a gradual increase that was paralleled by a boost in Th17 percentage at week 12 (Figure 1b).

The only difference observed between pCR and pPR within HER2-positive patients was a significant increase in the percentage of Treg cells at diagnosis in patients who experienced a pCR. However, both pCR and pPR groups showed a progressive enlargement of this CD4^+^ T cell subset that was compensated by an enhanced pool of Th17 cells at week 12 only in pCR patients. The number of B cells significantly decreased in partial responders, whereas the CD4/CD8 ratio significantly heightened in pCR patients. Interestingly, TP treatment induced a significant increase in the number of NK independently from pathological response (Figure 1c).

### Trastuzumab and Paclitaxel NC positively modulated innate immunity by boosting NK cells efficiency in HER2-positive patients

**Figure 2:**
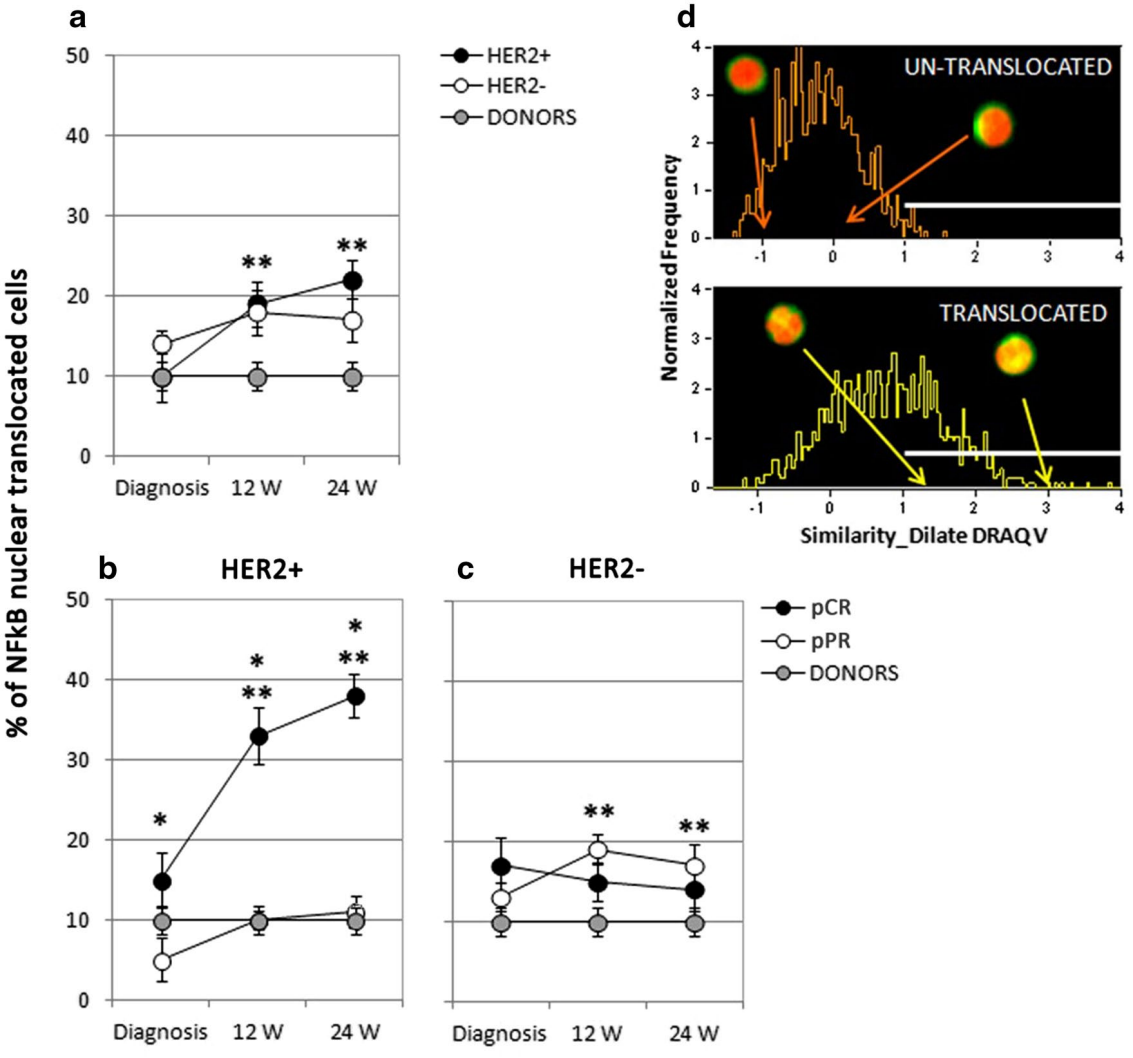
NF-kB nuclear translocation in NK cells of HER2-positive and HER2-negative patients throughout NC. **a** Quantification of the nuclear translocation of NF-kB in NK cells of HER2-positive (HER2+; *black dots*; n = 12) and HER2-negative patients (HER2−; *white dots*; n = 10) at diagnosis and during NC, and in healthy donors (DONORS; *gray dots*; n = 10). **b** Analysis performed within HER2-positive patients comparing NF-kB nuclear translocation observed in NK cells of patients undergoing a pathological complete response (pCR, *black dots*; n = 5) with that measured in case of partial response (pPR, *white dots*; n = 7) throughout NC, and with levels found in healthy donors (*gray dots*; n = 10). *p < 0.05 comparing values observed in case of complete response with those measured in partial responders. **c** Comparison of NF-kB nuclear translocation observed in the NK cells of HER2-negative patients achieving a pathological Complete response (pCR, *black dots*; n = 3) with levels measured in NK cells of HER2-negative partial responders (pPR, *white dots*; n = 7) during NC, and with the amount noticed in NK cells from healthy donors (*gray dots*; n = 10). **d** Representative plots obtained by Multispectral Flow Cytometry showing typical histograms of NK cells without (*upper plot*) or with (*lower plot*) the nuclear translocation of NF-kB. Representative cell pictures are shown. *W* week, *pCR* pathological complete response, *pPR* pathological partial response; **p < 0.05 in respect to the corresponding diagnosis values.

Within the innate immunity compartment, we considered the pivotal role of NK cells in anti-tumor immunity and their contribution to the induction of clinical responses to drugs acting through immune-mediate mechanisms, such as Trastuzumab [16]. To investigate the role of NK cells in mediating the response to NC, we better characterized the functionality of this cell subset by investigating the activation of the NF-κB transcription factor by Multispectral Flow Cytometry. This technique allows a precise enumeration of cells carrying a nuclear translocation of the p65 component of the complex, a marker of NF-κB activation (Figure 2d).

No differences in the percentage of NK cells with a nuclear translocation of p65 were highlighted between HER2-positive and HER2-negative patients at diagnosis and throughout NC treatment (Figure 2a). HER2-positive patients disclosed an increased percentage of cells with the NF-κB nuclear translocation during NC if compared to diagnosis levels (Figure 2a), whilst in the HER2-negative cohort this increase was evident only in patients undergoing a partial response (Figure 2c). Interestingly, within the HER2-positive arm, patients achieving a pCR displayed an enhanced percentage of NK cells characterized by NF-κB activation if compared to patients undergoing a partial response at diagnosis and throughout NC treatment, and in addition, diagnosis levels significantly increased during therapy only in case of pCR (Figure 2b).

NK cells are considered one of the main effectors triggering Trastuzumab-mediated ADCC, a mechanism of action that seems to actively contribute to the induction of a clinical response to this drug [19]. We therefore run an in vitro ADCC assay using patients’ PBMCs as effectors, and assessed the efficiency of Trastuzumab-dependent ADCC lysis after normalization for NK cell numbers (10,000). This analysis was performed in patients affected by a HER2-overexpressing cancer and treated with Trastuzumab and taxol, and in a control group of age-matched healthy women. At diagnosis, both patients achieving a pCR and those characterized by a partial response showed lower ADCC levels compared to healthy donors (not significant). However, pCR patients had a slightly higher ADCC efficiency than partial responders (Table 1). The analysis of ADCC levels performed along NC treatment and compared to diagnosis values revealed a decreased ADCC efficiency (ratio <1) in both groups of patients. Nevertheless, while partial responders revealed a continuous decrease of normalized ADCC values till the end of treatment, in pCR cases we noticed an opposite trend after 12 weeks characterized by slightly enhanced ADCC levels (Table 1). Due to the wide variability of ADCC levels measured through this in vitro assay, we highlighted no significant differences between the two groups of patients.

**Table 1:** In vitro Trastuzumab-mediated ADCC efficacy during NC in HER2-positive patients

		Diagnosis	12° W	24° W
		Normalized values	Ratio	Ratio
		% Lysis/10,000 NK	% Normalized lysis 12°W/% normalized lysis diagnosis	% Normalized lysis 24°W/% normalized lysis diagnosis
Healthy women (*n* = 20)	Mean ± SD (min–max)	23.13 ± 18.15 (5.06–90.68)		
pCR patients (*n* = 14)	Mean ± SD (min–max)	20.83 ± 18.00 (4.33–72.54)	0.52 ± 0.34 (0.15–1.26)	0.65 ± 0.38 (0.03–1.43)
pPR patients (*n* = 21)	Mean ± SD (min–max)	16.63 ± 14.98 (0.00–46.70)	0.72 ± 0.38 (0.18–1.54)	0.58 ± 0.47 (0.00–1.25)

*NK* Natural Killer cells, *W* week, *SD* standard deviation, *pCR* pathological complete response, *pPR* pathological partial response.

### A more functional anti-tumor adaptive immunity is maintained throughout NC in patients achieving a pCR compared to partial responders, especially in the HER2-positive arm

**Figure 3:**
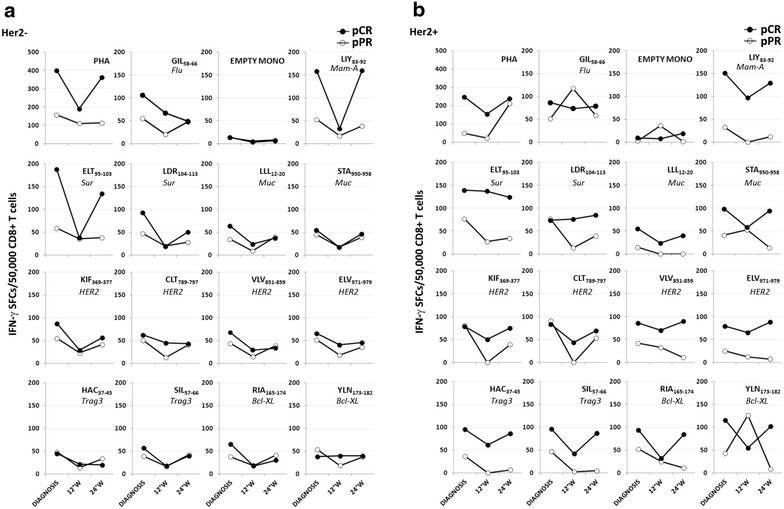
CD8^+^ T cell responses to BC-associated epitopes assessed by IFN-γ-ELISPOT in BC patients throughout NC. **a** Quantification of CD8^+^ T cell responses against 13 HLA-A*0201-restricted epitopes derived from 6 different BC-associated antigens (or against the flu matrix protein1-derived epitope) at diagnosis and during NC (12° and 24° week) in HER2-negative patients achieving a complete response (pCR, *black dots*; n = 2) and in those characterized by a partial response (pPR, *white dots*; n = 5). PHA-treated T cells and empty monocytes (EMPTY MONO) were used as positive and negative controls, respectively. **b** Analysis of CD8^+^ T cell responses against TAA-derived epitopes in HER2-positive patients reaching a complete response (pCR, *black dots*; n = 4) and a partial response (pPR, *white dots*; n = 2). *SFC* Spot Forming Cells, *pCR* pathological complete response, *pPR* pathological partial response, *PHA* phytohemagglutinin, *Flu* influenza, *Mam-A* Mammaglobin-A, *Sur* survivin, *Muc* mucin-1, *Trag3* taxol resistance associated gene 3, *W* week.

Pre-existing T-cell responses against TAA have been reported in patients with solid tumors and BC, in particular. At diagnosis, we observed that the ability to stimulate the generation of anti-tumor CD8^+^ T cells seemed to be more pronounced in HER2^+^ cancers [23]. We thus hypothesized that adaptive immunity might have a role in the response to NC, especially in the HER2-positive group of patients. Characterization of adaptive anti-tumor immune responses in our cohorts of patients was carried out by IFN-γ ELISPOT assay of spontaneous CD8^+^ T cell responses to 13 HLA-A*0201-restricted peptides derived from 6 different TAA (Mammaglobin-A, Survivin, Muc-1, HER2, Trag-3, Bcl-xL) [28–34] in 6 HER2-positive and 7 HER2-negative patients expressing the HLA-A*0201 allele. CD8^+^ T cell responses against all these epitopes were detectable in both groups of patients throughout treatment, even if a generalized decrease in the amount of these responses was usually observed after 12 weeks of treatment (Figure 3).

Interestingly, HER2-negative patients achieving a pCR (n = 2) showed a marked increase in the number of IFN-γ-producing CD8^+^ T cells only after stimulation with Mammaglobin-A and Survivin-derived peptides if compared to partial responders (n = 5), both at diagnosis and at the end of treatment (Figure 3a).

The same analysis performed in the HER2-positive arm revealed that, compared to partial responders (n = 2), patients undergoing a pCR (n = 4), displayed at diagnosis higher numbers of CD8^+^ T cells specific for all six TAAs investigated, in response to at least one of the derived epitopes (Figure 3b). Moreover, the prevalence of TAA-specific T cell responses in pCR patients was usually maintained throughout NC (Figure 3b). Interestingly, immunohistochemical analysis carried out in two representative cases revealed a higher infiltration of CD8^+^ cells in the tumor microenvironment of a patient achieving a pCR compared to a partial responder (Additional file 4). Intriguingly, while TAA-specific T-cell responses were always detectable in pCR patients, after 12 weeks of treatment partial responders showed no detectable CD8^+^ T-cell responses against some of the Mammaglobin-A, Muc-1, Her2-, and Trag3-derived epitopes (Figure 3b).

**Figure 4:**
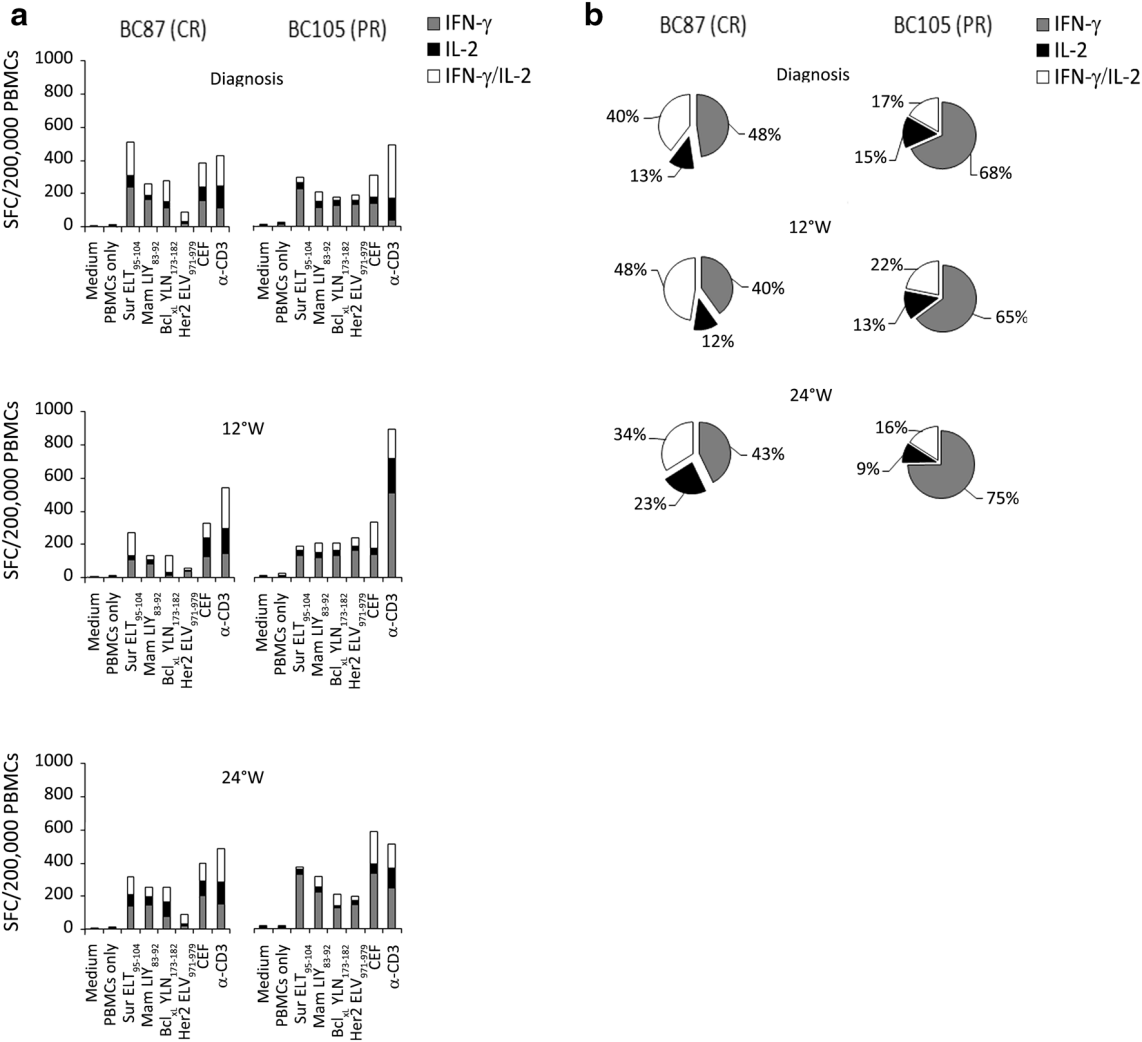
Polyfunctional CD8^+^ T cell responses to BC-associated antigens assessed by DualSpot in HER2-positive patients during NC. **a** Representative histograms of the quantification of IFN-γ (*gray bars*), IL-2 (*black bars*), and IFN-γ/IL-2 (*white bars*) releasing CD8^+^ T cells after stimulation with four epitopes derived from four different BC-associated antigens obtained at diagnosis and during NC in one HER2-positive patient achieving a pathological complete response (BC87, pCR) and in one HER2-positive patient characterized by a partial response (BC105, pPR). Medium and PBMCs alone were used as negative controls, CEF and α–CD3 as positive controls. **b** Slices of cakes represent the means of the percentages of CD8+ T cells releasing IFN-γ (*gray*), IL-2 (*black*), or IFN-γ/IL-2 (*white*) after stimulation with all the four epitopes derived from BC-associated antigens in case of complete response (*left*) or partial response (*right*). *SFC* spot forming cells, *pCR* pathological complete response, *pPR* pathological partial response, *W* week, *Sur* survivin, *Mam* mammaglobin-A, *CEF* mix of peptides derived from Cytomegalovirus, Epstein-Barr virus, and influenza.

To examine more in depth the functionality of TAA-specific T-cells, we further investigated the simultaneous release of IFN-γ and IL-2 after stimulation with TAA-derived peptides. The analysis was carried out for 4 selected single epitopes (Mammaglobin-A LIY_83-92_, Survivin ELT_95-104_, Her2/neu ELV_971-979_, Bcl-xL YLN_173-182_) (Figure 4a) and also considering together the T-cell responses against all the 4 different TAAs (Figure 4b). This latter evaluation revealed that, in the HER2-positive arm, the relative percentage of CD8^+^ T cells releasing both cytokines in response to TAA-specific peptides was higher in patients achieving a pCR (n = 2) compared to partial responders (n = 2), at the expense of single-cytokine producing cells (Figure 4).

## Discussion

The results of the present study extend our previous findings demonstrating that, at diagnosis, patients with locally advanced, HER2-overexpressing BC are characterized by a retained immune proficiency [23], and more importantly, achieve a high rate of pCR (42.5%) after neoadjuvant Trastuzumab and Paclitaxel [35]. These observations, together with the known immune-modulating effects of these drugs [36, 37], prompted us to thoroughly investigate the immune profile of the same cohort of patients along treatment. The time frame in which NC is administered is particularly suitable for immunological studies [36] because it does not suffer from the immune suppression induced by surgery or by advanced metastatic disease.

We herein demonstrate that, in both groups of patients, NC treatment induced significant changes in the relative proportions of circulating immune cells. We first examined what happened within the innate immune compartment, with particular attention to NK cells, given the major role of these immune cells in mediating the therapeutic effects of monoclonal antibodies such as Trastuzumab. NK cells showed an opposite behaviour in the two cohorts of patients, suggesting a different involvement of the innate responses in these two different tumor subtypes. We have previously shown that, at diagnosis, the HER2-negative group had a higher percentage of circulating NK cells than HER2-positive cases [23]. This NK cells prevalence was mainly found in HER2-negative partial responders, suggesting that this change is functionally ineffective, being probably a consequence of the concomitant reduction in the T cell compartment. This altered balance between the NK and T cell pools could underlie an impairment of adaptive anti-tumor immune responses in HER2-negative patients unable to achieve a pCR. Nevertheless, we observed that the ED NC regimen induced a partial restoration of the balance between NK and T cells and was associated with an increased number of activated NK cells carrying nuclear translocation of NF-κB in partial responders, probably caused by the DNA-damage response induced by anthracyclines, able to alert the innate immune system against the tumor [36].

In contrast, HER2-positive patients showed progressively increased NK cell percentages both in cases undergoing pCR and in partial responders, without evidence of concomitant alterations in the number of circulating T cells. Recent data convincingly demonstrated that taxanes are able to enhance T cell and NK cell functions [36, 38], and in vitro experiments showed that Paclitaxel enhanced the cytotoxicity of human NK cells by increasing perforin production through NF-κB activation [39]. We herein showed a progressive and significant increase in the number of NK cells with activated NF-κB in HER2-positive patients, and in particular in those undergoing a pCR, suggesting a direct role of Paclitaxel treatment in mediating this effect. Intriguingly, the efficiency of Trastuzumab-dependent ADCC, mainly due to NK lytic efficiency [15], at diagnosis was higher in pCR patients if compared to partial responders, and at the end of NC, pCR patients showed a slight recovery of normalized ADCC lysis, whereas partial responders disclosed an opposite trend. However, the differences between the two groups of patients did not reach the statistical significance probably due to the broad variability of ADCC activity, already observed through this kind of experiments [40]. Larger series are thus required to establish whether the efficiency of Trastuzumab-dependent ADCC may constitute a marker predictive of pCR induction. However, the investigation of the innate immune compartment revealed a major role of NK cells in the cohort of patients treated with Trastuzumab, underlying a potential association between the increased activation state of these cells and the induction of a pCR.

Interestingly, Trastuzumab may also indirectly elicit a specific adaptive immune response, in particular against the HER2 TAA, by stimulating HER2-specific cytotoxic T cells through enhanced HER2 degradation, internalization, and presentation by MHC-class I molecules [5, 37]. We thus quantified the amount of T cell responses specific for different epitopes derived from the HER2 protein during Trastuzumab-including NC. Although no increase in the number of HER2-specific T cell responses was observed in the time frame considered by this study, HER2-positive patients undergoing pCR, but not partial responders, maintained throughout NC a high prevalence of T cells specific for the same two HER2 epitopes recognized at higher levels at diagnosis. This suggests that retained T-cell responses against HER2 may favor the induction of a pCR in patients treated with Trastuzumab.

We then investigated the presence of anti-tumor adaptive immunity against other TAA usually expressed by different BC histotypes. One of the most relevant findings of the present study is the correlation between enhanced T cell responses against epitopes derived from Mammaglobin-A and Survivin, detectable till the end of NC therapy, and the induction of pCR in both HER2- positive and -negative subgroups. These results are consistent with a prevalent polarization of T-cell responses towards these antigens in BC, irrespective to the HER2 status of the tumor. Consistently with an enhanced immunogenicity of HER2-positive BC, patients with HER2-overexpressing tumors achieving a pCR showed an increased number of IFN-γ producing T cells after stimulation with peptides derived from a broader range of TAAs, which included in addition to HER2, Mammaglobin-A, and Survivin, also Muc-1, Trag3, and Bcl-XL. Notably, a higher prevalence of TAA-specific T cells producing both IFN-γ and IL-2 was observed in these patients, thus suggesting a major clinical role of the multi-epitopic and polyfunctional anti-tumor T cell responses. In both groups of patients, TAA-specific T-cell responses decreased after the first 12 weeks of NC. This is probably due to an initial immunosuppressive effect of NC also favored by the corticosteroids premedication usually associated with taxane administration. Moreover, in any of the two groups of treatment we noticed a boosting of the anti-tumor T cell response during NC, even if both anthracyclines and taxanes have been associated with the induction of immunogenic features [38, 41]. It can be argued that the time frame of NC is perhaps too short to observe an increase in peripheral anti-tumor T cell responses, particularly those generated de novo. Intriguingly, the increased proportion of peripheral naïve T cells, observed at the end of NC among CD8^+^ T cells in the HER2-positive arm, may constitute a potential reservoir of T cell precursors that may be primed at the tumor site, thus leading to the de novo generation of anti-tumor T cells. In addition, a representative immunohistochemistry analysis in two HER2-positive patients revealed a higher infiltration of CD8^+^ cells within the tumor microenvironment in case of pCR. Further in situ analyses may better investigate the presence of an effective and increased immune response after NC, as described in other studies [8]. Finally, we are currently performing the same analysis in samples collected 2 months after surgery and at annual follow up, to assess whether anti-tumor T-cell responses measured during NC are lasting over time and able to generate immunological memory. This investigation will clarify the potential prognostic role of an effective anti-tumor adaptive immunity in BC patients undergoing NC.

The efficiency of anti-tumor T cells may be widely influenced by several immunosuppressive mechanisms developed by the tumor itself or induced in the host through the expansion of immunosuppressive cell subsets. With regard to Treg cells, several clinical studies revealed that the depletion of circulating Treg induced by cyclophosphamide-based NC reflected within the tumor microenvironment [11], and their in situ absence after NC was associated with higher pCR rates in BC patients [42, 43]. In both our cohorts of patients after cyclophosphamide-free NC we observed an increase in circulating Treg percentages that may have contributed to inhibit the boosting of anti-tumor T cell responses. This observation strongly encourages to carefully consider the immune-modulation properties of conventional chemotherapy when designing the NC schedule [13], especially in the presence of Trastuzumab. The stimulation of anti-tumor T cell responses promoted by this antibody may indeed benefit from the inhibition of tumor-induced immune suppression [18, 44].

Trastuzumab is also able to disrupt the balance between Treg and Th17, enhancing Th17 percentages during treatment and reducing Treg compartment in case of metastasis [22]. Our data interestingly highlighted a significant increase in the proportion of Th17 cells after 12 weeks of treatment only in case of pCR, thus supporting the involvement of the Treg/Th17 ratio in mediating the response to NC in this setting.

In addition, intratumor and circulating Th17 cells were associated with improved prognosis and clinical response to therapy in BC [11, 45] and in vivo data suggested the occurrence of a strict cooperation between Th17 cells and CD8^+^ cytotoxic T lymphocytes in mediating effective anti-tumor immune responses [46]. In our analysis, especially in HER2-positive patients achieving a pCR, we noticed a significant increase in the number of Th17 cells at week 12 of NC treatment that was usually followed (at week 24) by an enhancement of TAA-specific CD8^+^ T cell responses.

## Conclusions

In conclusion, our data strongly support the relevance of immune-related factors in mediating the response of BC patients to NC treatment [18], and the independence of immunological parameters from classical predictive markers [13]. The integrated immunomonitoring performed in this study revealed that the peripheral immune function may constitute a valid source of suitable predictive biomarkers in the NC setting. In particular, in HER2-positive patients treated with Trastuzumab and taxanes, the assessment of NK cell activation and functionality throughout NC may become a strategy to identify patients more likely achieving a pCR. The analysis of adaptive anti-tumor T-cell responses against selected TAAs may instead constitute a powerful tool to identify, since the time of diagnosis, which patients will reach a pCR after NC in both HER2-negative and HER2-positive background. Finally, the increased percentage of Tregs, balanced by the Th17 enhancement only early on during the NC treatment, prompts to consider alternative combinations in the NC schedule that favor the reversion of cancer-related immune suppression, strengthening host anti-tumor immune responses and increasing the likelihood to reach a pCR. The preliminary results obtained with the present study may thus favor the identification of relevant immune biomarkers, whose validation is warranted by further prospective clinical trials.

## Additional files

Additional file 1: Flow cytometry gating strategy. Representative flow cytometry plots showing the gating strategy used to identify immune cell subsets. SS, Side Scatter; FS, Forward Scatter; CM, Central memory; EM, effector memory, Temra, terminally differentiated; NK, natural killer; Treg, regulatory T cells; Th17, T helper 17 cells. Additional file 2: Exemplary ELISPOT pictures derived from survivin-, Flu/CEF-, or un-stimulated T cells. Representative wells obtained by IFN-γ ELISPOT assay (panel A) or by IFN-γ/IL-2 dual color ELISPOT assay (panel B) in HER2-positive breast cancer patients. **A.** Triplicates of wells obtained after stimulation of CD8^+^ T cells thorugh monocytes loaded with ELT_95-103_ survivin-derived peptide (upper line), GIL_58-66_ influenza-derived peptide (middle line), no peptides (lower line), at diagnosis (left triplicates) and after 24 weeks (right triplicates) of neoadjuvant chemotherapy in a patient undergoing a pathological complete response (left panel) and in a case of pathological partial response (right panel). **B.** Triplicates of wells achieved after stimulation of PBMCs with ELT_95-103_ survivin-derived peptide (upper line), a mix of CMV-EBV-Flu-derived peptides (middle line), no peptide, at diagnosis and after 24 weeks of neoadjuvant chemotherapy in a case of pathological complete response (left panel) and in a patient undergoing a pathological partial response. Sur, survivin; mono, monocytes; W, week. Additional file 3: Memory phenotyping of CD4^+^ and CD8^+^ T cells during NC in HER2-negative and HER2-positive patients. Comparison of the percentages of memory cell subsets within CD4^+^ and CD8^+^ T cells in HER2-negative (n=25) and HER2-positive (n=16) patients at diagnosis and during NC. W, week; *p<0.05 comparing values obtained in HER2-positive with those observed in HER2-negative patients. **p<0.05 in respect to the corresponding diagnosis levels. Additional file 4: Immunohistochemistry analysis of lymphocyte infiltration in the tumor microenvironment. Two selected HER2-positive cases achieving pCR and pathological partial response, respectively, were analyzed by immunohistochemistry to characterize the presence of CD8+ cells within tumor microenvironment. Specimens were routinely fixed in 10% buffered formalin, embedded in paraffin and then stained with H&E for histological examination (upper panels). For immunohistochemical analyses (lower panels), 2 to 3 μm serial sections of primary tumors were processed with automated immunostainer Benchmark XT (Ventana, Tucson, AZ, USA), and staining was carried out with CD8 (clone SP57, Ventana Medical System, Tucson, AZ, USA) diluted 1:100. Nuclear counterstaining was accomplished with Harris’ hematoxylin. Omission of the primary antibody was used as a negative control. Representative microscopic fields were showed at 10x original magnification.

## Abbreviations

ADCC: antibody-dependent cell cytotoxicity
APCs: antigen presenting cells
BC: breast cancer
Bcl-XL: B-cell lymphoma-extra large
BSA: bovine serum albumine
CEF: cytomegalovirus, Epstein-Barr virus, influenza virus
CISH: chromogenic in situ hybridization
CMV: cytomegalovirus
Cy: cyanine
EBV: Epstein-Barr virus
ECD: phycoerythrin-texasred
ED: epirubicin docetaxel
ELISPOT: enzyme-linked immunosorbent spot
FBS: fetal bovine serum
FISH: fluorescence in situ hybridization
FITC: fluorescein isothiocyanate
HBSS: Hank’s Balanced Salt Solution
HER2: human epidermal growth factor receptor 2
IFN: interferon
IHC: immunohistochemistry
IL: interleukin
moAb: monoclonal antibody
muc-1: mucin 1
NC: neoadjuvant chemotherapy
NK: natural killer cell
PBMCs: peripheral blood mononuclear cells
PBS: phosphate-buffered saline
pCR: pathological complete response
PE: phycoerythrin
PMA: phorbol 12-myristate 13-acetate
pPR: pathological partial response
SS: similarity score
TAA: tumor-associated antigen
T_CM_: central memory T cells
T_EM_: effector memory T cells
T_naive_: naive T cells
TP: trastuzumab Paclitaxel
T_Temra_: terminally differentiated T cells
Th: T helper cell
TIL: tumor infiltrating lymphocytes
TN: triple negative
Trag-3: taxol-resistance associated gene 3
Treg: regulatory T cell
ULN: upper limit of normal
