# Supplementary figures and images for: Improved Natural Killer cell activity and retained anti-tumor CD8+ T cell responses contribute to the induction of a pathological complete response in HER2-positive breast cancer patients undergoing neoadjuvant chemotherapy
Source: J Transl Med. 2015 Jun 27;13:204. doi: 10.1186/s12967-015-0567-0 (PMC4483222; doi:10.1186/s12967-015-0567-0)

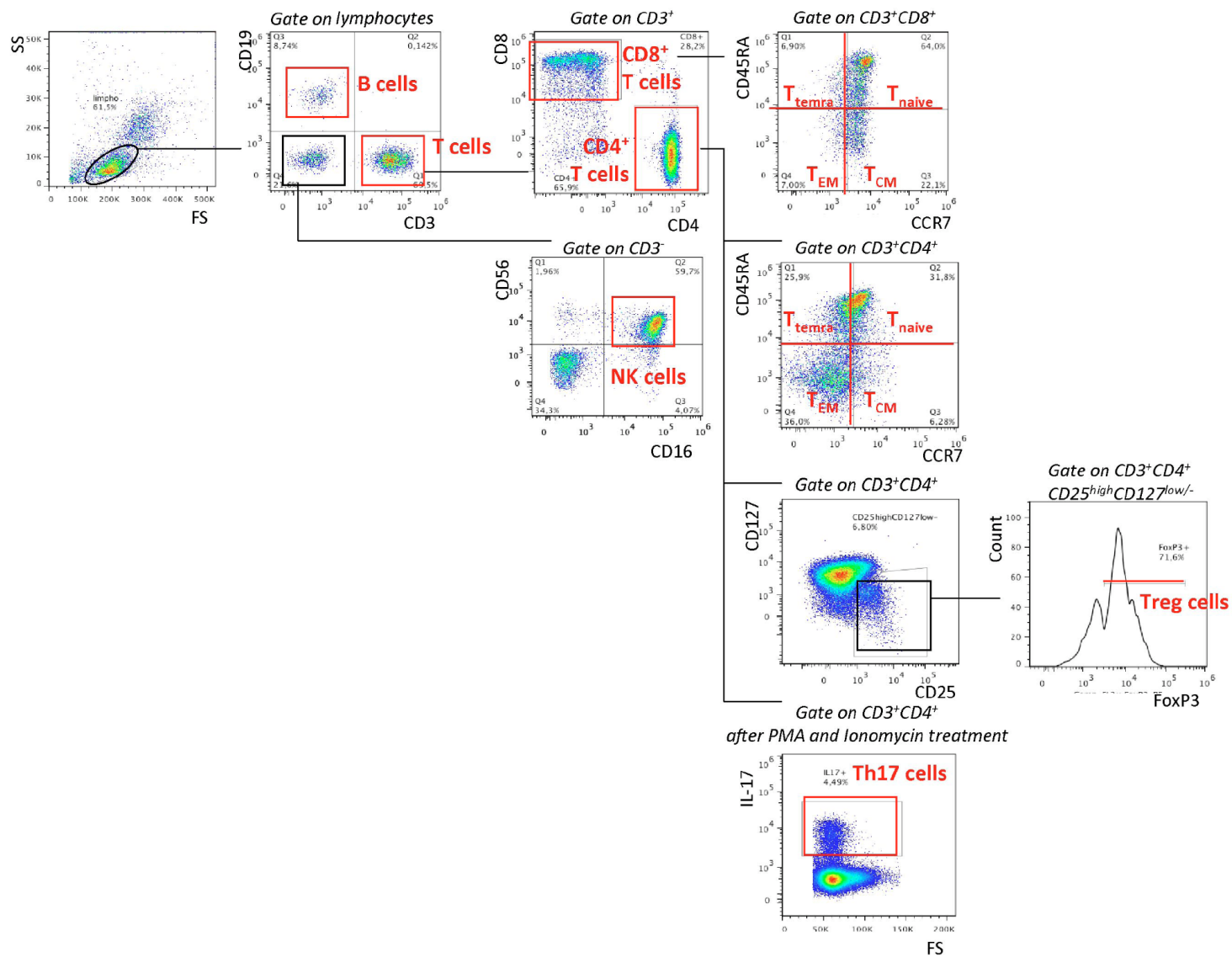

Supplement: Additional file 1: — Flow cytometry gating strategy. Representative flow cytometry plots showing the gating strategy used to identify immune cell subsets. SS, Side Scatter; FS, Forward Scatter; CM, Central memory; EM, effector memory, Temra, terminally differentiated; NK, natural killer; Treg, regulatory T cells; Th17, T helper 17 cells. [file 12967_2015_567_MOESM1_ESM.pdf]

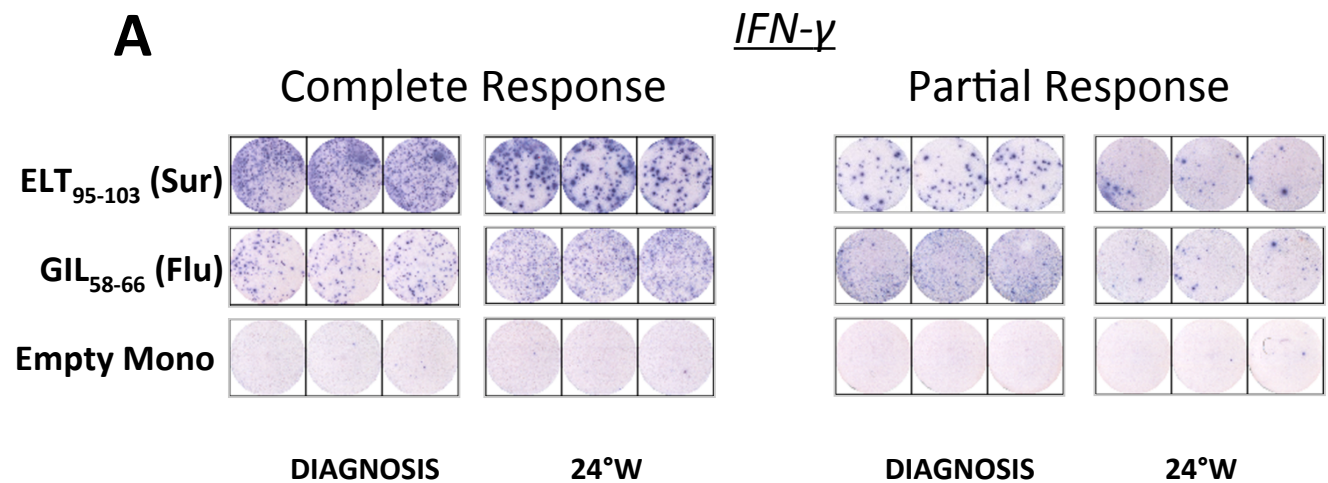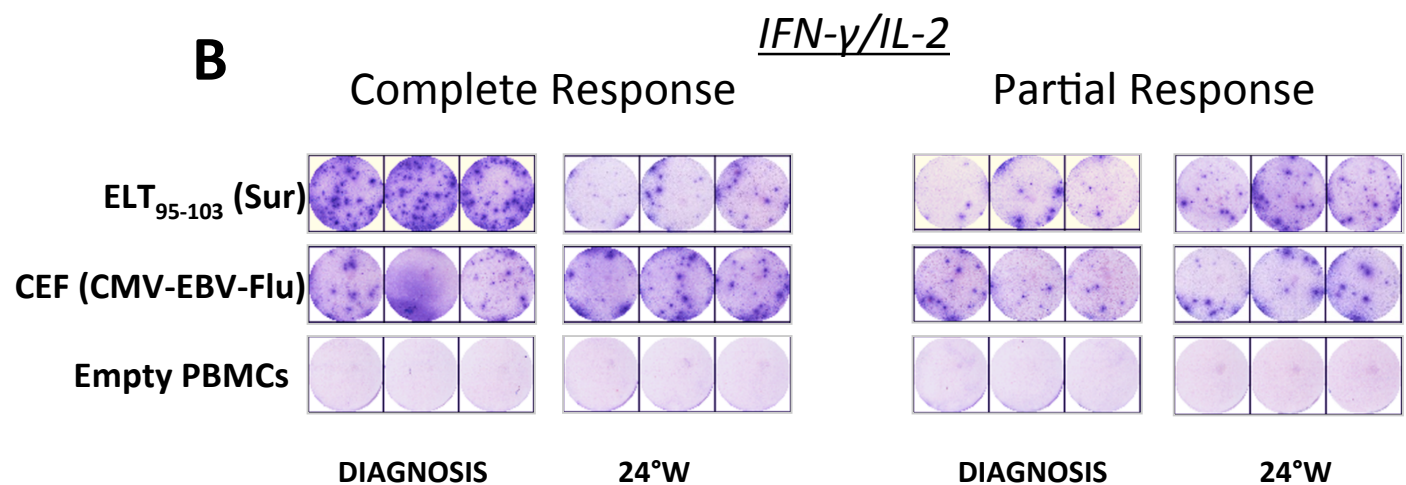

Supplement: Additional file 2: — Exemplary ELISPOT pictures derived from survivin-, Flu/CEF-, or un-stimulated T cells. Representative wells obtained by IFN-γ ELISPOT assay (panel A) or by IFN-γ/IL-2 dual color ELISPOT assay (panel B) in HER2-positive breast cancer patients. A. Triplicates of wells obtained after stimulation of CD8+ T cells thorugh monocytes loaded with ELT95-103 survivin-derived peptide (upper line), GIL58-66 influenza-derived peptide (middle line), no peptides (lower line), at diagnosis (left triplicates) and after 24 weeks (right triplicates) of neoadjuvant chemotherapy in a patient undergoing a pathological complete response (left panel) and in a case of pathological partial response (right panel). B. Triplicates of wells achieved after stimulation of PBMCs with ELT95-103 survivin-derived peptide (upper line), a mix of CMV-EBV-Flu-derived peptides (middle line), no peptide, at diagnosis and after 24 weeks of neoadjuvant chemotherapy in a case of pathological complete response (left panel) and in a patient undergoing a pathological partial response. Sur, survivin; mono, monocytes; W, week. [file 12967_2015_567_MOESM2_ESM.pdf]

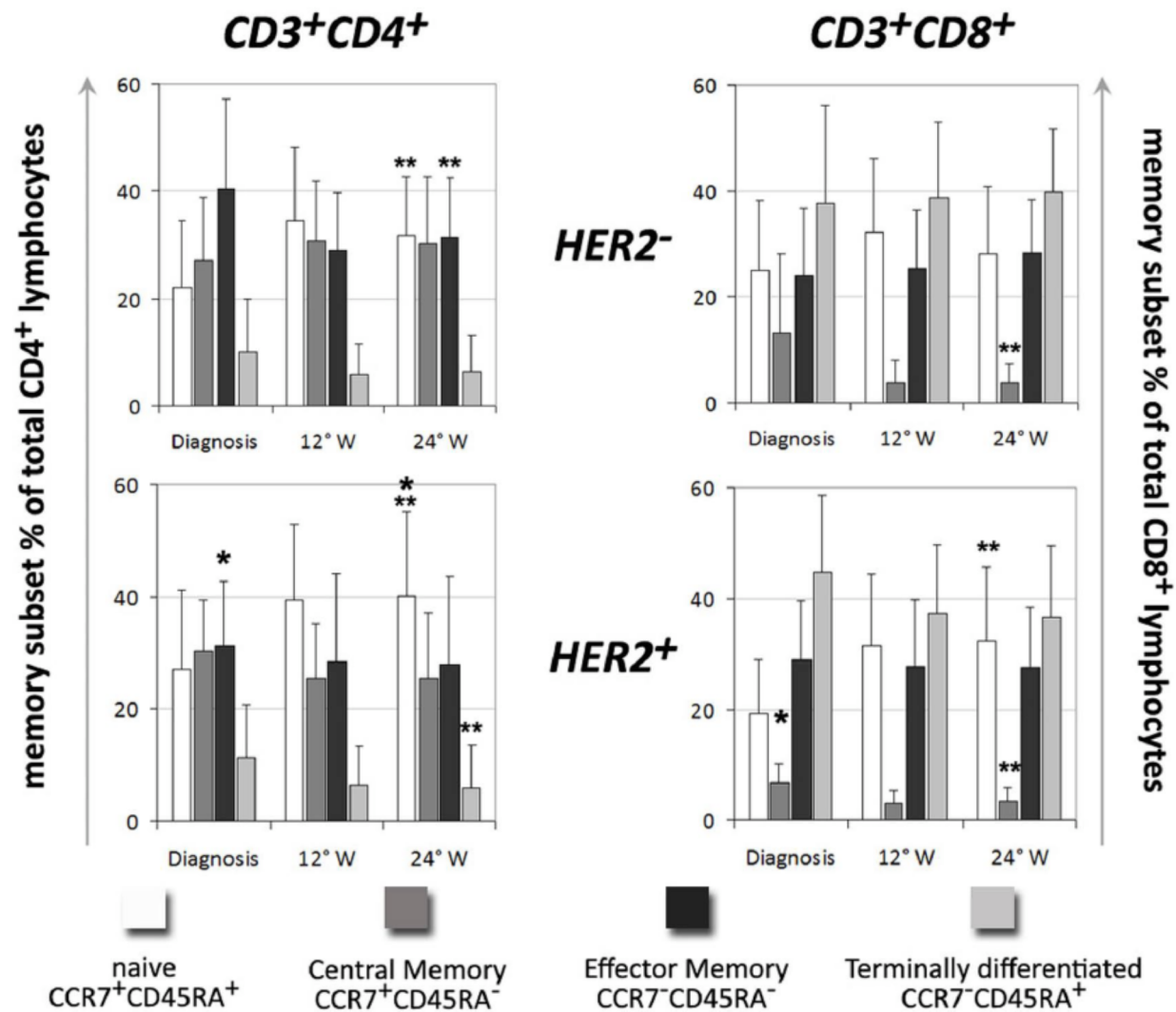

Supplement: Additional file 3: — Memory phenotyping of CD4+ and CD8+ T cells during NC in HER2-negative and HER2-positive patients. Comparison of the percentages of memory cell subsets within CD4+ and CD8+ T cells in HER2-negative (n=25) and HER2-positive (n=16) patients at diagnosis and during NC. W, week; *p<0.05 comparing values obtained in HER2-positive with those observed in HER2-negative patients. **p<0.05 in respect to the corresponding diagnosis levels. [file 12967_2015_567_MOESM3_ESM.pdf]

**pathological Complete Response**

**pathological Partial Response**

**H&E**

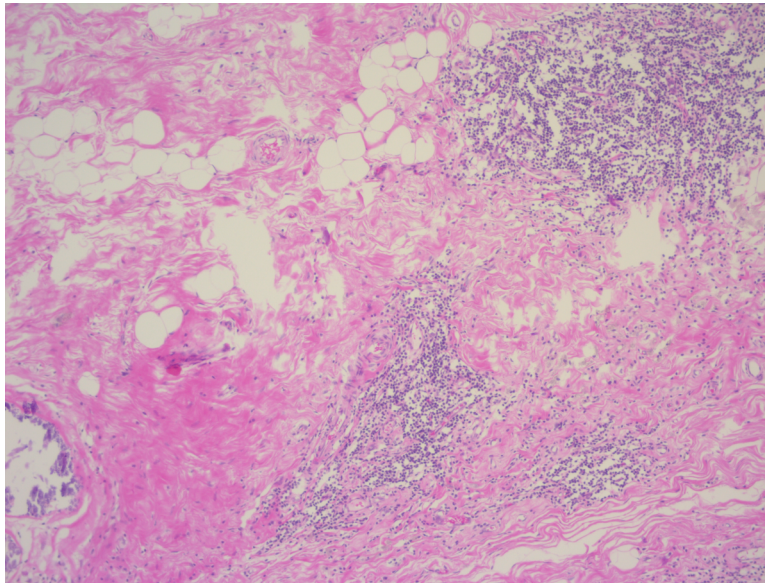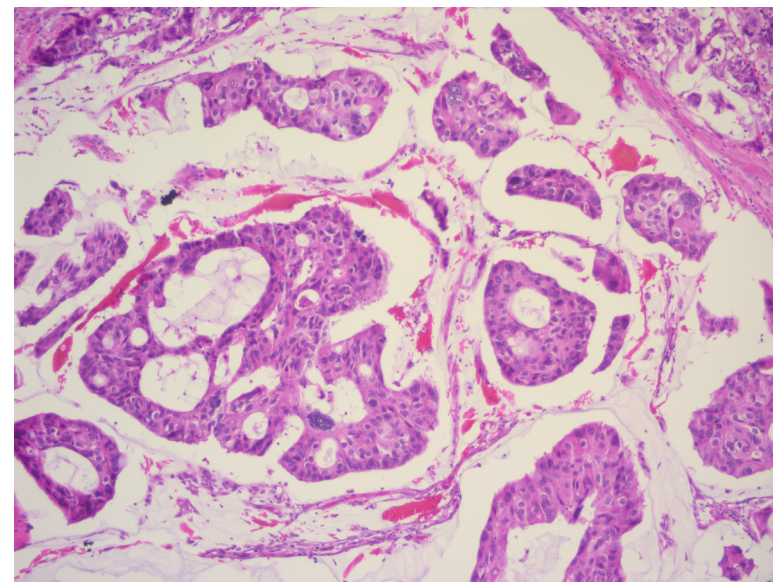

**CD8**

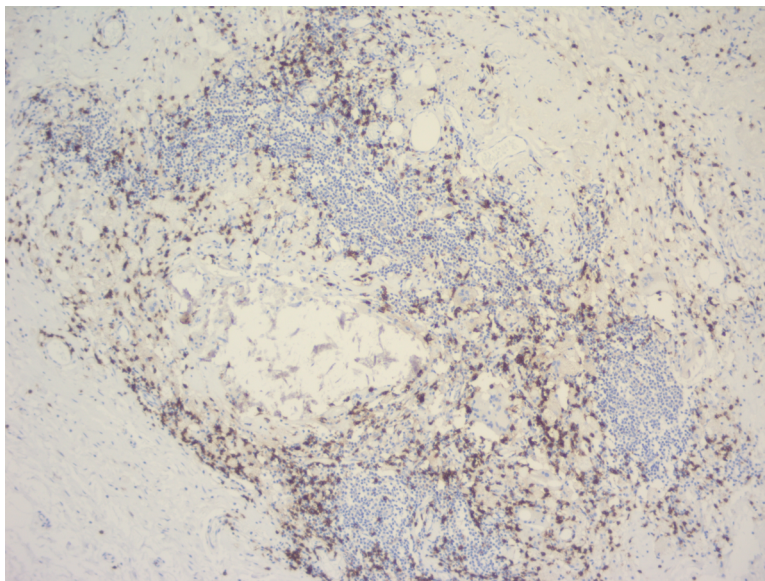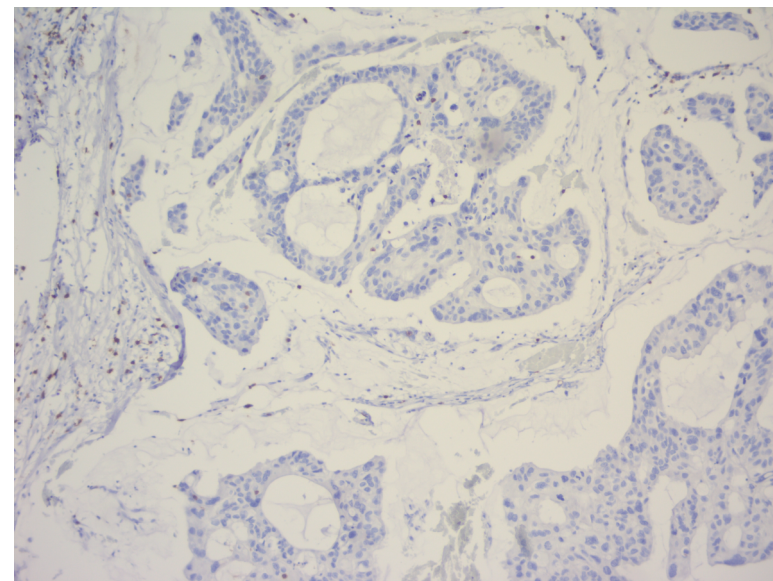

Supplement: Additional file 4: — Immunohistochemistry analysis of lymphocyte infiltration in the tumor microenvironment. Two selected HER2-positive cases achieving pCR and pathological partial response, respectively, were analyzed by immunohistochemistry to characterize the presence of CD8+ cells within tumor microenvironment. Specimens were routinely fixed in 10% buffered formalin, embedded in paraffin and then stained with H&E for histological examination (upper panels). For immunohistochemical analyses (lower panels), 2 to 3 μm serial sections of primary tumors were processed with automated immunostainer Benchmark XT (Ventana, Tucson, AZ, USA), and staining was carried out with CD8 (clone SP57, Ventana Medical System, Tucson, AZ, USA) diluted 1:100. Nuclear counterstaining was accomplished with Harris’ hematoxylin. Omission of the primary antibody was used as a negative control. Representative microscopic fields were showed at 10x original magnification. [file 12967_2015_567_MOESM4_ESM.pdf]
